# In situ measurements of dissolved gases in xylem sap as tracers in plant physiology

**DOI:** 10.1093/treephys/tpae062

**Published:** 2024-06-10

**Authors:** Capucine Marion, Mana Gharun, Matthias S Brennwald, Rolf Kipfer

**Affiliations:** Department of Water Resources and Drinking Water EAWAG, Swiss Federal Institute of Aquatic Science and Technology, Ueberlandstrasse 133, 8600 Dübendorf, Switzerland; Institute of Biogeochemistry and Pollutant Dynamics, Swiss Federal Institute of Technology (ETH), Universitätstrasse 16, 8092 Zürich, Switzerland; Department of Geosciences, Institute of Landscape Ecology, University of Münster, Schlosspl. 2, 48149 Münster, Germany; Department of Water Resources and Drinking Water EAWAG, Swiss Federal Institute of Aquatic Science and Technology, Ueberlandstrasse 133, 8600 Dübendorf, Switzerland; Department of Water Resources and Drinking Water EAWAG, Swiss Federal Institute of Aquatic Science and Technology, Ueberlandstrasse 133, 8600 Dübendorf, Switzerland; Institute of Biogeochemistry and Pollutant Dynamics, Swiss Federal Institute of Technology (ETH), Universitätstrasse 16, 8092 Zürich, Switzerland; Institute of Geochemistry and Petrology, Swiss Federal Institute of Technology (ETH), Clausiusstrasse 25, 8092 Zürich, Switzerland

**Keywords:** continuous measurements, gas transport in plants, in situ probes, in vivo measurement, mass spectrometry, root water uptake, sap velocity estimation, tracer experiment

## Abstract

Trees transport gases from the ground into the atmosphere through the process of transpiration. Tracing gases transported through this mechanism continuously and under field conditions remains an experimental challenge. Here we measured gases dissolved in the tree sap in situ and in real time, aiming to simultaneously analyse the transport of several gases (He, Ar, Kr, N_2_, O_2_ and CO_2_) from the soil, through the trees, into the atmosphere. We constructed and inserted custom-made semi-permeable membrane probes in the xylem of a fir tree and measured gas abundances at different heights using a portable gas equilibrium membrane-inlet mass spectrometer (‘miniRUEDI’). With this method, we were able to continuously measure the abundances of He, Ar, Kr, N_2_, O_2_ and CO_2_ in sap over several weeks. We observed diurnal variations of CO_2_ and O_2_ concentrations that reflected tree physiological activities. As a proof of the concept that trees do uptake dissolved gases in soil water, we irrigated the tree with He-enriched water in a tracer experiment and were able to determine upward sap flow velocity. Measurements of inert gases together with reactive species, such as CO_2_ and O_2_, allowed separation of the physical transport and exchange of gases derived from the soil or atmosphere from biological reactions. We discuss the opportunities that our technique provides for continuous in situ measurements of gases in the tree sap.

## Introduction

Soil, plants and atmosphere are connected through water fluxes driven by water potential gradients between the soil and the atmosphere. During transpiration, plants take up soil water and release it into the atmosphere through evaporation from the stomatal cavity on the surface of their leaves. Depending on the local climate, species and efficiency of their water use, plants can transpire considerably large amounts of water (tens of litres per day, Nobel 2020) during carbon uptake and growing period, making water fluxes via the soil–plant–atmosphere continuum the largest water flux from the Earth’s terrestrial surface (Bernacchi and Vanloocke 2015). Improving the understanding of plant water uptake, plant water source and water transport in plants is crucial not only to understand plant physiology but also to constrain the global hydrological cycle and link below-ground geochemical processes with atmospheric chemistry.

It is commonly observed that in contrast to surface water, soil and groundwater contain concentrations of N_2_, and noble gases exceeding those in air-saturated water (ASW, e.g. water being at solubility equilibrium with the free atmosphere). Atmospheric gases are found to be in excess of 10% to 50% in the soil water (Kipfer et al. 2002). The specific (or intentionally modified for tracer test purposes) noble gas signature of groundwater is often used by hydrogeologists to better understand the spatial and temporal evolution of groundwater flow, to constrain water residence time and to reconstruct environmental conditions at recharge (Kipfer et al. 2002; Brennwald et al. 2013, 2022; Giroud et al. 2023).

Tall plants, through high transpiration rates, relocate water from deeper soil layers into the atmosphere. Even though the general transport process of water through trees is well understood (McElrone et al. 2013; Pfautsch 2016), hardly anything is known about how and to which extent water-dissolved gases are transferred by plants from the soil and groundwater to the atmosphere.

The well-established concepts of terrestrial noble gas geochemistry provide ideal tools to investigate such physical processes in plants and might add to a better understanding of the plant-mediated exchange of water and gases between the biosphere and the atmosphere. Adaptation of these hydrogeological methods to plant studies could provide experimental techniques to study the transport and dynamics of dissolved gas species in plant sap.

However, there are not yet adequate analytical techniques available for in situ (noble) gas analysis in plants, and accordingly, gas studies in plants are very scarce. Bushong investigated the gas composition of a cottonwood tree for the first time in 1907 (Bushong 1907). Since then, few studies have addressed gas concentrations in the tree sap and their dynamics, and none was ever conducted to measure such variables in situ or performed continuous measurements. Such continuous in situ measurements are key in environmental studies as they sidestep destructive sampling, reduce setup bias, enable the study of systems under natural conditions, and capture short- and long-term dynamics and responses (e.g. due to changes in climate).

Schenk et al. (2016) studied dissolved atmospheric gases in the xylem sap of a red trumpet vine (*Distictis buccinatoria*) through off-line membrane inlet mass spectroscopy (MIMS). Ar and N_2_ were found to be clearly supersaturated compared to atmospheric equilibrium (Schenk et al. 2016), but the authors did not identify groundwater and excess air as a potential source of this measured gas supersaturation. To our knowledge, this paper remains the only study that attempted to measure dissolved gases in xylem sap. Megonigal et al. (2020) analysed radon (^222^Rn) emissions for three different tree species (*Fagus grandifolia*, *Liriodendron tulipfera* and *Quercus rubra*) and showed that Rn is indeed transported and emitted by trees (Megonigal et al. 2020). Although not pointed out by the authors, the trees’ Rn emissions were correlated to their rooting depth. Deeper root systems access deeper groundwater, commonly enriched with Rn being in radioactive equilibrium with the soil matrix. This observation proves that trees allow soil gases to emanate into the atmosphere, and it strengthens our hypothesis that at least part of the gases in the tree sap originate from soil water gas that is taken up by the roots. After reviewing the existing literature on gases in trees, it appears that there is no available method to measure soil or groundwater-derived gases in situ in the sap.

**Figure 1:**
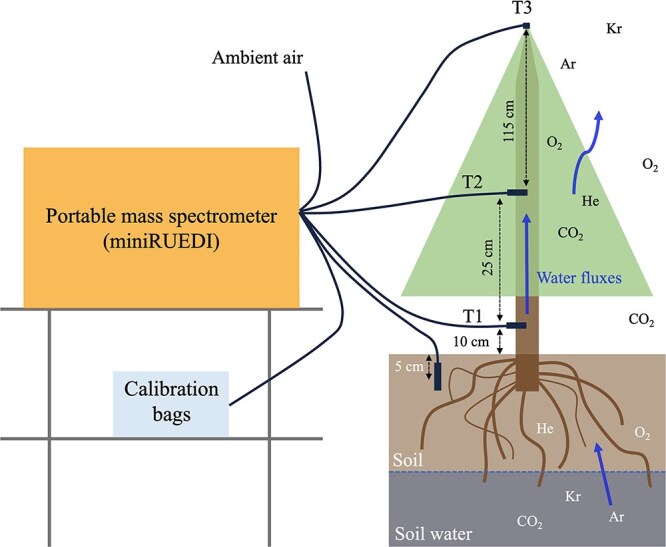
Experimental setup to analyse gases in trees and conceptual overview of gas exchange within a tree’s environment. Membrane probes to separate gas and liquid phases were installed in the soil and inside the tree xylem of an *A. alba* at about 10 cm (T1) and 35 cm (T2) height and at the tip of the tree (T3). The membrane probes were connected to a gas-tight capillary to a portable mass-spectrometer (‘miniRUEDI’) to continuously measure the concentrations of noble gases; unresponsive to the tree physiological activities (He, Ar and Kr); and O_2_ and CO_2_ to monitor physiological gas exchange (Maechler et al. 2012; Brennwald et al. 2016; Gasometrix GmbH n.d.). The measurements were calibrated with the use of two tailored standards (see Tables S1 and S2 available as Supplementary data at *Tree Physiology* online).

We designed and tested a novel, but still rough-and-ready, in situ technique to continuously determine dissolved gases in xylem water and in soil (Fig. 1). By adapting methods used for quasi-continuous gas analysis in hydrogeological systems, we developed a setup for trees enabling continuous measurements, with a time resolution better than 1 h. The method also renders it possible to carry out tracer experiments, whereby noble gases (e.g. artificially added) are used to track water uptake by trees.

In the first trials, we investigated if atmospheric (noble) gases could be determined in xylem sap and if a soil water gas supersaturation could be traced back to the sap. We addressed fluid transport in xylem only because it is the vascular system responsible for upward water transport in trees. Gas composition in xylary sap is hypothesized to be determined by the gas composition of the soil water utilized by the tree (Fig. 1).

## Materials and methods

### Experimental setup

For our experiment, we chose an *Abies alba Mill.* tree of roughly 150 cm in height and a basal stem diameter of 4.5 cm. The tree was kept in a pot inside a climate chamber (for additional information, see Table S3 available as Supplementary data at *Tree Physiology* online). Climate chamber conditions (temperature, light and humidity) were controlled as described in Table 1 to sustain sufficient transpiration during the study period. Figure 1 shows the experimental setup, whose technical details and analytical procedures are described in the following sections.

**Table 1:** Climate chamber settings.

Time (h)	Temperature (°C)	RH (%)	Light intensity (kLux)
06:00–18:00	24	60	32
18:00–06:00	14	60	0

### Mass spectrometer

The ‘miniRUEDI’ (Gasometrix GmbH n.d., Switzerland) is a portable mass spectrometer able to quantify the partial pressures of He, Ar, Kr, N_2_, O_2_, CO_2_, CH_4_, etc., in a gas mixture (Maechler et al. 2012; Brennwald et al. 2016; Gasometrix GmbH n.d.). The instrument has been designed to perform efficient on-site measurements of dissolved gases in various terrestrial fluids (Brennwald et al. 2016). Gases are continuously sampled and transferred through a capillary pressure reduction system into a vacuum chamber, where they are analysed with a quadrupole mass spectrometer (time resolution <1 min). The system operates at low gas consumption (<0.1 mL min^−1^). All technical specifications can be found in reference (Brennwald et al. 2016). The mass spectrometer measurements were calibrated using two standard gas mixtures stored in gas-tight bags (see Tables S1 and S2 available as Supplementary data at *Tree Physiology* online).

### Developed in situ membrane probes

**Figure 2:**
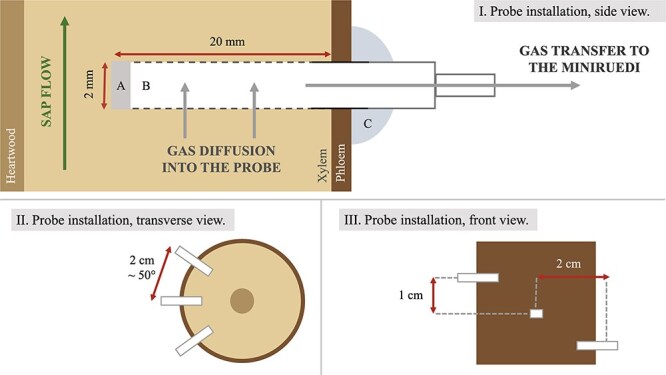
Installation of the membrane probes, schematic. I. Side view. (A) The inner end of the probe is sealed with silicone. (B) The probe tubing consists of polypropylene of outer diameter 2.0 mm and is ~20 mm long. Gas migrates through the probe to the inlet of the ‘miniRUEDI’ mass spectrometer. (C) Outside of the tree, the probe is sealed from the atmosphere with silicone rubber. II. View from above; each probe was installed radially 2 cm apart. III. Front view, each probe was separated by 1 cm height. A picture of sampling height T1 is provided in the Supplementary material (see Fig. S2 available as Supplementary data at *Tree Physiology* online).

We adapted our probes from a design by Volkmann and Weiler (2014) and a prototype used to analyse dissolved gases in pore water sediments (Engelhardt 2023). Our probes consist of a porous hydrophobic tubing acting as a semipermeable membrane between the surrounding xylem water (outside the probe) and a capillary inside the tube for gas sampling. Gases dissolved in xylem water diffuse through the membrane into the tube, while liquid water is blocked from entering the probe by the hydrophobic material. We used polypropylene tubing generously provided by 3M™ (PP S6/2 Capillary Membrane). Gases in the probes were directly sampled through a capillary connected to the mass spectrometer (Fig. 2).

The probes were designed to have a small outer diameter (2 mm) to minimize damage to the studied tree. Three individual probes were combined into a single array to ensure a larger sampled gas volume needed for reliable and robust gas analysis by the mass spectrometer. A second type of probe using similar tubing but with a larger diameter (6 mm) was used for soil gas analysis, allowing gas determination from a single larger probe (see Table S4 available as Supplementary data at *Tree Physiology* online, and Figures S1 and S2 available as Supplementary data at *Tree Physiology* online).

### Probe installation

Two days before the entire experimental setup was installed, holes were drilled into the tree stem for probe installation. The holes were flushed with acetone to prevent resin formation and wound sealing (Marshall et al. 2020). Since acetone is relatively volatile, it quickly evaporated from the tree. We did not notice any reaction from the tree to this treatment. Immediately before probe installation, the holes were flushed again with acetone. Each hole had a diameter equivalent to the outer diameter of the probe (2 mm) and an approximate depth of 2.5 cm (see Fig. 2).

In response to the drilling, xylem tracheids opened to the atmosphere and got air-filled. Since xylem cells operate under tension, probe installation probably disturbed physiological operation in adjacent cells (e.g. xylem cells opened to the atmosphere, causing the gas-meniscus inside the tracheids to pull back, and altering sap flow around these cells, Pfautsch 2016). Even if probe installation might have impeded normal sap flow around the probes, gas diffusion into the probe still allows us to measure gas concentrations at the sampling points. Indeed, tracheids are connected both axially and radially to other conducting cells, so the measured gas phase should be in contact with the sap-filled tracheids through pits.

### Climate chamber

The climate chamber allowed control over temperature, light conditions and to a certain extent also relative humidity. Specific settings used during the experiment are given in Table 1. Before measurements started, the tree was placed in the climate chamber for ~2 week to adapt to its new environmental setting.

### Tracer experiment

For the tracer experiment, plastic bags were installed around the pot of the tree to isolate the source of the tracer (the soil) from the sampling ports above the soil (e.g. along the stem or in the ambient air) and to reduce the risk of cross-contamination. Then, we irrigated the tree with helium-spiked water, through a silicone tube inserted into the soil. After irrigation, the tube was removed, and the sealing bags were tightly closed around the stem. To prevent anoxic conditions around the roots, the bags were removed after 5 days to allow oxygen inflow in the root zone (Glenz et al. 2006; see Figure S3 available as Supplementary data at *Tree Physiology* online).

### Data analysis

The mass-spectrometric signals of the analysed gas species (or their fragments) were recorded as peak intensities at *m/z* = 4 (He), 15 (CH_4_), 28 (N_2_), 32 (O_2_), 40 (Ar) and 44 (CO_2_), as described in Brennwald et al. (2016). Note that, in contrast to the GE-MIMS technique (Brennwald et al. 2016), the gas/sap exchange in the membrane probes did not attain solubility equilibrium, which impedes accurate quantification of the respective partial pressures according to the GE-MIMS technique. Instead, for the purposes of this work, we report the ratios of the peak intensity *I_k_* of each gas (*k* = He, CH_4_, N_2_, O_2_, CO_2_) to that of Ar. These ratios were further normalized to the corresponding peak-intensity ratios determined in the standard gas (see Tables S1 and S2 available as Supplementary data at *Tree Physiology* online) used to calibrate the gas analysis:


$$ {R}_k=\frac{{\left({I}_k/{I}_{\mathrm{Ar}}\right)}_{\mathrm{sample}}}{{\left({I}_k/{I}_{\mathrm{Ar}}\right)}_{\mathrm{standard}}}. $$

Note that the normalization of the Ar ion current in *R_k_* compensates for the effects of physical processes controlling the dynamics of Ar and other inert gases in the plant. With the exception of residual effects from species-dependent fractionation controlled by diffusion or gas exchange, variations in *R_k_* therefore mainly reflect physiological processes in the plant.

**Figure 3:**
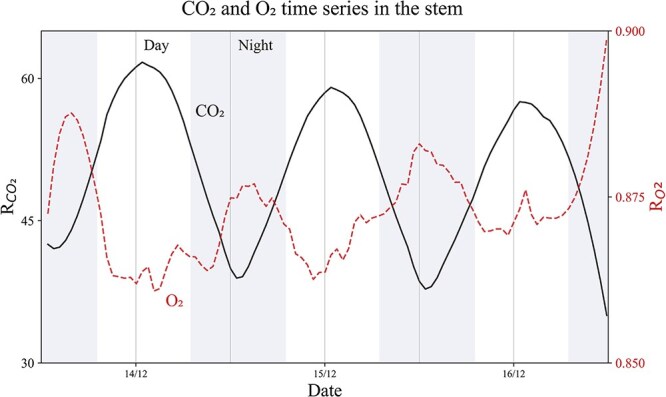
The normalized ion currents for ratio CO_2_ and O_2_ to Ar in the stem. Day and night periods of the measuring interval (*x*-axis) are indicated with shades of grey and refer to the climate chamber setup (Table 1). Stem measurement refer to the mean of all measured tree heights (see Fig. 1). The mean error on *R*_CO2_ is <2% and mean error on R_O2_ is <1%.

When whole stem measurements are discussed, they are reported as the average of tree heights T1, T2 and T3. Time series measurements (e.g. in Fig. 3) were smoothed with a Savitzky–Golay filter with a window length of 20 points and a third-order polynomial function.

## Results

Testing of our experimental setup lasted from 6 December 2021 until 27 January 2022, and a tracer experiment with helium was conducted on 14 January 2022. Maintenance and optimization of the experimental setup led to interruption of the measurements. Thus, in this section, we will only concentrate on a few days of continuous measurements.

### Noble and reactive gas time series

We observed variation in *R*_CO2_ (peak intensities at *m*/*z* = 44; see Materials and methods, Data analysis) in the stem to oscillate between 35 and 60, peaking during the day and reaching its lowest levels at night. *R*_CO2_ peaked just after noon and started increasing again after midnight. In contrast, *R*_O2_ in the stem peaked during the night and oscillated between 0.86 and 0.9 times (Fig. 3). The values of *R*_CO2_ were an order of magnitude greater than those of *R*_O2_, and the *R*_CO2_ time series was smoother than that of *R*_O2_.

### Tracer experiment

Using the same experimental setup, we performed a tracer experiment, during which we watered the tree with helium-spiked water, aiming to determine the propagation of helium signal along the path of sap ascending within the tree.

**Figure 4:**
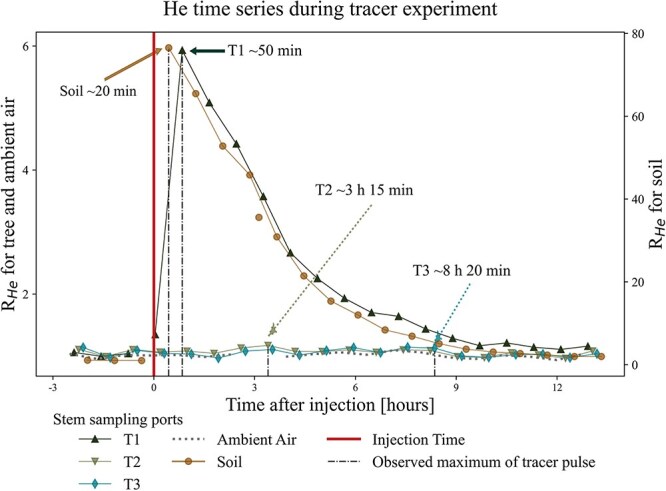
Time series of the normalized ion currents for ratio of He to Ar during the tracer experiment. Time after injection in hours is given on the horizontal axis. Both vertical axes display *R*_He_, with different scales for tree/ambient air and soil values. The vertical line at 0 marks the injection time when the tree was irrigated with He-labeled water. Maximums in *R*_He_ signal are marked by arrows (dashed for less statistically different maximas), and travel times of the tracer are indicated. See Figure 1 for the positioning of the different sampled heights.

The breakthrough of He was clearly detected in the soil and at the sampling port T1 (Fig. 4). The first measurements in the soil and at T1 were taken, respectively, 20 and 50 min after the injection, and were the highest measured He signal at these sampling heights. The tracer breakthrough for tree heights T2 and T3 can also be observed; *R*_He_ maxima occurred after 3 h 15 min (T2) and 8 h 20 min (T3, see Fig. 4). However, these peak values at T2 and T3 are strictly speaking not statistically significant (falling inside a confidence interval of 99% for normally distributed datasets).

We noted that *R*_He_ in the soil was about one order of magnitude higher than in the tree, and no tracer enrichment was observed in the ambient air of the climate chamber.

From the distance between the sampling ports (Fig. 1) and the time delay between the different *R*_He_ maxima, we determined sap velocity. Table 2 presents the different sap velocities derived from the distance between the sampling ports and the respective tracer travel times. The time interval between subsequent measurements was around 8 min, and the same sampling point was measured approximately every 45 min. Thus, our estimation of the sap velocity is a rough approximation. The mean sap velocity derived from this tracer experiment was around 20 cm h^−1^.

**Table 2:** Sap velocity calculations.

Sampling ports	Distance (cm)	Tracer travel time (h)	Sap velocity (cm h^−1^)
Soil – T1	15	0.5	30
Soil – T2	40	3	13
Soil – T3	155	8	20
T1 – T2	25	2.5	10
T2 – T3	115	5	23
Overall mean	ND	ND	19

## Discussion

These two measurement sets hold the proof of concept that our experimental setup can effectively determine gas dynamics in trees in situ. They also demonstrate that the application of tracer hydrological methods, as commonly used in groundwater research, can successfully be applied to assess fluid exchange and transport in trees.

### Noble gas and reactive gas time series

The observed diurnal variations in *R*_CO2_ and *R*_O2_ (Fig. 3) are the direct observation of the tree respiration. The observed increase in *R*_CO2_ matches the photosynthesis active hours, going along with a decrease in *R*_O2_. At night, *R*_CO2_ decreases and *R*_O2_ rises due to increased O_2_ uptake from respiration. Similar stem CO_2_ variations were observed in another study on *Picea abies* (Etzold et al. 2013).

The fact that the relative amplitude of *R*_CO2_ variations is larger than that of *R*_O2_ can be explained by the higher O_2_ background in atmospheric air (around 21% of atmospheric air composition, Ozima and Podosek 2002). In contrast, atmospheric air contains ~400 p.p.m. of CO_2_. This low atmospheric background allows a better resolution of tree-induced CO_2_ variations as compared with those of O_2_. Thus, the difference of one order of magnitude between the *R*_CO2_ and the *R*_O2_ time series is attributed to this normalization of atmospheric ratios.

Since noble gases are inert, variations in their concentrations cannot be caused by metabolic processes in trees, in the soil, or in the groundwater. Atmospheric inert gases (i.e. He, Ar, Kr and N_2_) are naturally present in soil water and are used to trace the physical exchange processes of gases in soil water (Kipfer et al. 2002; Aeschbach-Hertig and Solomon 2013; Brennwald et al. 2013). Thus, the simultaneous measurement of inert gases (e.g. He, Ar, Kr and N_2_) with gases central to tree metabolism (CO_2_ or O_2_) allows separation of physical gas transport processes in trees from the chemical processes ensuing from tree metabolic activities. The normalization of reactive gas to a non-reactive gas species, virtually removes all transport and exchange processes, solely related to physical mechanisms (gas/water partitioning or diffusion may still have small effects on the results if the two studied gas species have different Henry/diffusion coefficients). Thus, *R*_CO2_ and *R*_O2_ presented in Figure 3 mainly illustrate gas exchange induced by biological reactions.

An in-depth analysis and interpretation of the produced data with regards to plant physiology would be outside the scope of this technical paper. However, with our results, showing in situ continuous time series of reactive gas species normalized by a noble gas within a tree, we aimed to show the technical feasibility of such measurements and paved the way for robust in situ gas analysis in tree physiology studies.

### Tracer experiment

The helium tracer injected into the soil water was observed to be transported along the sap ascent path. Thus, we confirmed our hypothesis that xylary gas composition is determined by dissolved soil gas composition.

Being very volatile, He is likely to have escaped from the soil water into the soil gas and accumulated in the plastic bag above the soil. Once out of the liquid phase, He was less accessible to the tree for uptake via the roots, and He degassing from the soil water probably happened faster than He uptake by the tree. This partitioning explains the order of magnitude of the difference in measured *R*_He_ between the soil and the tree. Additionally, some gas might be lost through diffusion through tree lenticels and the bark before the first measurement point in the tree (Carrillo-López and Yahia 2019). Such tracer loss through transpiration is particularly efficient at the height where needles are present (just above T1). Enhanced transpiration rates might explain why the measured *R*_He_ at T2 and T3 is much lower than at T1, as most of the tracer escaped through transpiring needles in between our measurement points.

As no He excess was observed in the ambient air of the climate chamber (Fig. 4), we conclude that most of the He was diluted within the climate chamber’s volume due to the active ventilation of the chamber.

The estimated mean sap velocity of 20 cm h^−1^ falls well in the range of expected sap velocities for *A. alba* (Wullschleger and King 2000). However, it should not be translated immediately to the whole-plant water transport velocity of mature trees. There can be large radial and azimuthal variations (within the sapwood cross section at the same height) in sap velocity with tree size, age and canopy leaf area (Wullschleger and King 2000; Shinohara et al. 2013). The interactions of canopy leaf area, stomatal regulation, sapwood area, with root depth and morphology balance the demand for water at the leaf level and supply through the stem, thus affecting the sap velocity. The fact that we measured clear tracer signals in a slow-transpiring tree indicates that our method works efficiently both for low- and high-transpiring trees.

### General comments on the experimental technique

To obtain real dissolved gas concentrations as results, instead of a ratio of gases (*R_k_*), we recommend strictly controlling temperature and pressure at every measurement point, which would allow the robust and reliable conversion of ion currents to gas partial pressures or concentrations.

Our interpretation of the results remains open in terms of tree physiology, as our work aimed to critically assess the technical feasibility of determining the in situ abondance of gases in the tree sap.

## Conclusion

Overall, the presented experimental technique is, to our knowledge, the first successful attempt in continuous measurement of dissolved gases in the tree sap and has the advantage of being noninvasive. While there is room for technical improvement, our method proved to be successful in providing evidence that gases can be used as tracers within trees, providing estimates of sap flow velocities and allowing the assessment of the dynamics of physiologically relevant gases for plant metabolism. The presented method can easily be applicable to studies on many plant physiological processes, such as water and gas uptake, gas diffusion, respiration, transpiration, photosynthesis, or embolism mechanisms and recovery.

## Supplementary Material

Marion_et_al_-Supplementary_Material_tpae062

## Data Availability

The discussed data are now under embargo before the publication of this study. It will be made available openly by Spring 2025.
