# Supplementary material for: In situ measurements of dissolved gases in xylem sap as tracers in plant physiology
Source: Tree Physiol. 2024 Jun 10;46(13):47–53. doi: 10.1093/treephys/tpae062 (PMC13016618; doi:10.1093/treephys/tpae062)
Supplement: Marion_et_al_-Supplementary_Material_tpae062 [file marion_et_al_-supplementary_material_tpae062.pdf]

**SUPPLEMENTARY MATERIAL – IN-SITU MEASUREMENTS OF DISSOLVED GASES IN XYLEM SAP AS TRACERS IN PLANT PHYSIOLOGY – Marion et al.**

*Table 1. Main gas standard composition: atmospheric air* concentrations *(Ozima and Podosek, 2023).*

| Gas species | Concentration [%] |
|---|---|
| $N_2$ | 78.10 |
| $O_2$ | 20.95 |
| Ar | 0.93 |
| He | $5.24 \times 10^{-4}$ |
| Kr | $6.50 \times 10^{-5}$ |

*Table 2. Second gas standard composition: atmospheric air enriched in $CH_4$ and $CO_2$.*

| Additional gas species compared to Table 1 | Concentration [%] |
|---|---|
| $CO_2$, $CH_4$ | 1.00 |

*Table 3. Soil composition.*

| Component | Fraction [%] |
|---|---|
| Land soil | 30 |
| Wood fiber | 15 |
| Compost | 15 |
| Coconut fiber | 10 |
| Bark humus | 10 |
| Black soil | 10 |
| Crushed expanded clay | 10 |

*Table 4. Dimensions of the various developed membrane probes.*

| | Inner diameter [mm] | Outer diameter [mm] | Length [mm] | Inner volume [mL] |
|---|---|---|---|---|
| Tree probe | 1.8 | 2.0 | 20 | 0.1 |
| Soil probe | 5.0 | 6.0 | 25 | 0.9 |

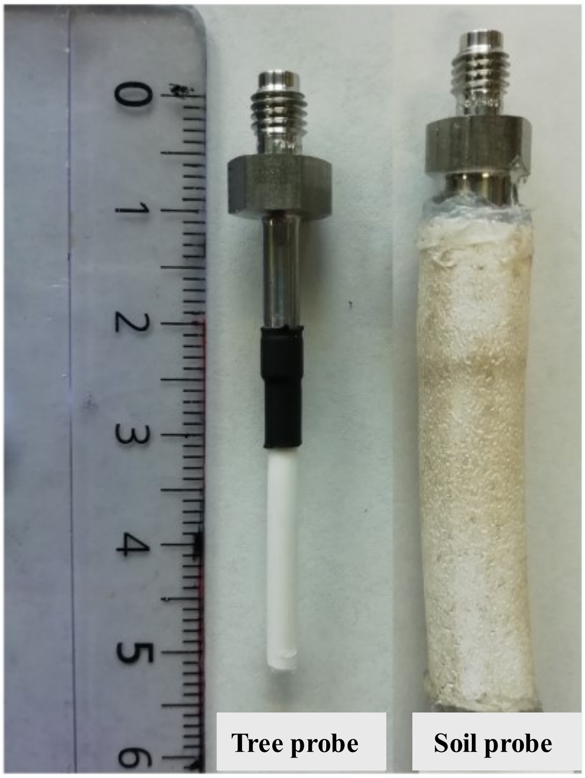


***Figure 1***. *Picture of the tree and soil probes.*

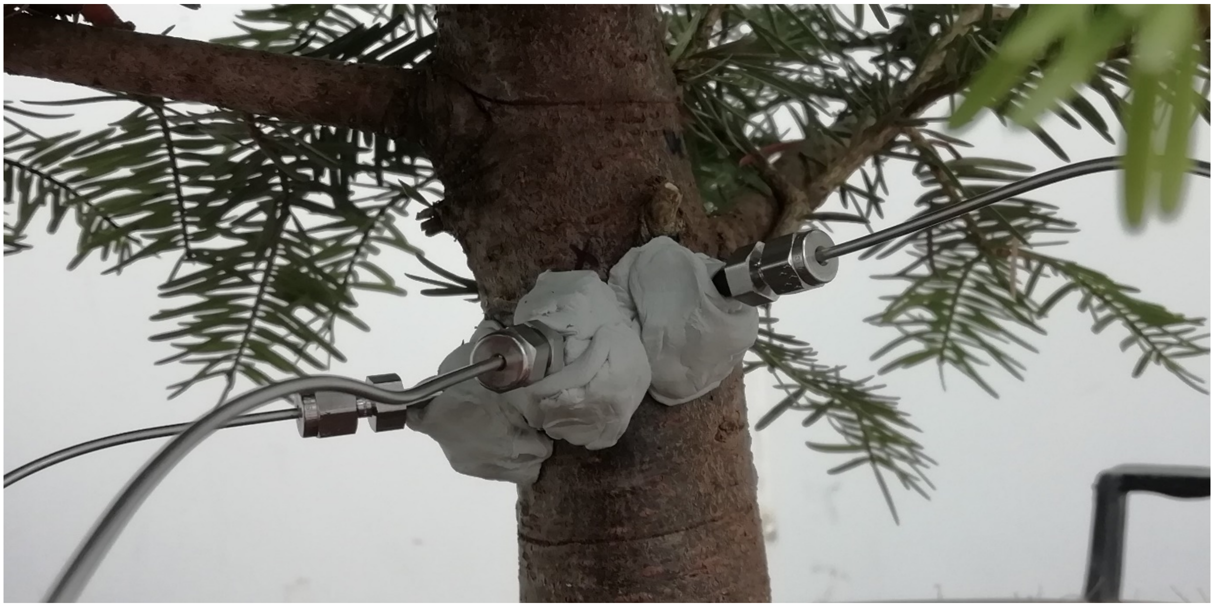

***Figure 2***. *At T1 and T2, 3 individual probes are combined to a single array to ensure a high enough gas flow for robust and stable gas detection. Here the array at T1 is shown.*

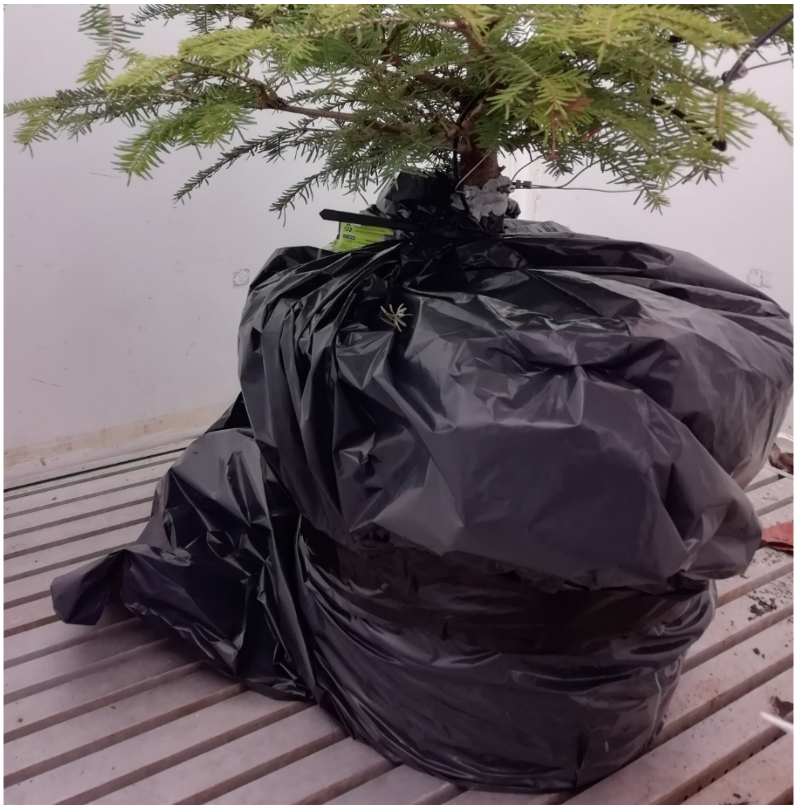

***Figure 3. Bagged pot during the tracer experiment.*** *For each gas tracer experiment, plastic bags were wrapped around the pot to isolate the soil from ambient of the climate chamber in order to minimize contamination.*

## REFERENCE:


- Ozima M. and Podosek F. (2002) Noble Gas Geochemistry. Cambridge University Press.
